# Association of plasma remnant cholesterol with cognitive function in the middle-aged and elderly Chinese adults with type 2 diabetes: a cross-sectional study

**DOI:** 10.3389/fnut.2026.1705243

**Published:** 2026-02-05

**Authors:** Xiuwen Ren, Chengjun Zhang, Zhi Duan, Sen Zhao, Zhihong Zhang, Xueying Zhang, Yu Sha, Lianyun Ju, Jie Mu, Yiyao Gu, Yunyun Gong, Linhong Yuan, Xinjing Guo, Ying Wang

**Affiliations:** 1 School of Public Health, Capital Medical University, Beijing, China; 2 Beijing Key Laboratory of Environment and Aging, Capital Medical University, Beijing, China; 3 Department of Pediatrics, Peking University First Hospital, Beijing, China; 4 Suzhou Research Center of Medical School, Suzhou Hospital, Affiliated Hospital of Medical School, Nanjing University, Suzhou, China; 5 Department of Hematology, Beijing Anzhen Hospital, Beijing, China; 6 Department of Nutrition and Food Hygiene, School of Public Health, Heinz Mehlhorn Academician Workstation, Hainan Medical University, Haikou, Hainan, China; 7 International Collaborative Research Center for the Development and Utilization of Tropical Food for Special Medical Purpose, Haikou, Hainan, China; 8 Jiangsu Province Engineering Research Center of Development and Translation of Key Technologies for Chronic Disease Prevention and Control, Suzhou Vocational Health College, Suzhou, China; 9 School of Food Science and Nutrition, University of Leeds, Leeds, United Kingdom

**Keywords:** dietary, middle-aged and older adults, mild cognitive impairment, remnant cholesterol, type 2 diabetes mellitus

## Abstract

**Background:**

The relationship between plasma remnant cholesterol (RC) level and cognitive function in middle-aged and older Chinese adults with type 2 diabetes (T2DM) was unclear.

**Methods:**

One thousand eight hundred seventeen participants aged 55 to 75 were recruited from communities in Beijing. Demographic information and daily dietary intakes were collected by self-designed questionnaire. Fasting venous blood was obtained for quantitative analysis of plasma lipid parameters. The Montreal Cognitive Assessment (MoCA) was used to assess cognitive function. To explore the association between plasma RC and the risk of mild cognitive impairment (MCI), we performed logistic regression analysis and restricted cubic spline (RCS). Additionally, subgroup analyses were conducted to assess the influence of potential co-founders on the association.

**Results:**

Plasma RC level was negatively correlated with daily intakes of vegetable, legume and fish intakes in patients with T2DM, and with daily intakes of cereals, vegetables, and legumes in non-T2DM subjects. Participants with plasma RC levels in the second (Q_2_), third (Q_3_), and fourth (Q_4_) quartiles had a higher risk for MCI compared to those in the first quartile (Q_1_) level of plasma RC, both in T2DM and non-T2DM participants. RCS results indicated a nonlinear relationship between plasma RC levels and the risk of MCI. Subgroup analysis showed that the association between plasma RC levels and the risk of MCI was pronounced in females and subjects aged 60 and above.

**Conclusion:**

An increase in plasma RC level is a potential risk factor for MCI. A plasma RC concentration below 0.578 mmol/L can decrease the risk of MCI in middle-aged and older individuals with T2DM. Similarly, a plasma RC concentration below 0.581 mmol/L may lower the risk of MCI in non-T2DM subjects. Consuming vegetables and legumes daily could help reduce the concentration of RC.

## Graphical abstract

**Figure:**
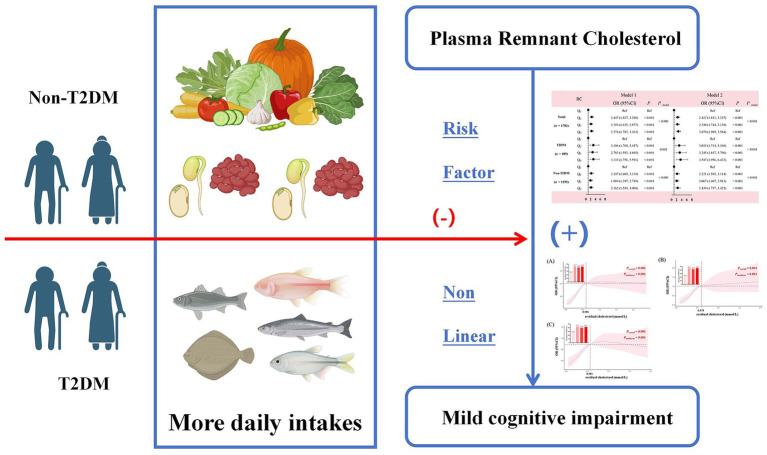
This graphical abstract illustrates the association between plasma remnant cholesterol (RC), and the risk of mild cognitive impairment (MCI) among middle-aged and older adults with and without type 2 diabetes mellitus (T2DM). Higher plasma RC levels are associated with an increased risk of MCI in both T2DM and non-T2DM populations, exhibiting a nonlinear dose-response relationship. Dietary patterns rich in vegetables, legumes, cereals, and fish are negatively correlated with plasma RC concentrations.

## 1 Background

Mild cognitive impairment (MCI) serves as a transitional stage between healthy brain aging and dementia, representing a critical pathological stage for initiating preventive treatment to delay the progress of dementia. Globally, the prevalence of MCI among community-dwelling individuals aged 50 and above has surpassed 15%, which indicating that the prevention and management of cognitive decline is a serious public health challenge (1).

Remnant Cholesterol (RC) refers to the total cholesterol (TC) remaining after subtracting high-density lipoprotein cholesterol (HDL-C) and low-density lipoprotein cholesterol (LDL-C), including cholesterol contained in very low-density lipoprotein (VLDL) and intermediate-density lipoprotein (IDL) particles that are hydrolyzed during fasting, as well as cholesterol in chylomicron residues in the postprandial state (2). RC level can be estimated using a formula or measured through techniques such as ultracentrifugation and nuclear magnetic resonance spectroscopy (3). RC is increasingly recognized as a direct factor in the development of atherosclerosis and as an additional marker of cardiovascular and cerebrovascular disease risk. Studies have shown that RC can remain in the artery, promote foam cell formation and inflammatory response, thereby accelerating the process of atherosclerosis (4). RC is also associated with atherosclerotic plaque instability and may increase the risk of ischemic stroke (5). In addition, abnormal RC metabolism may induce cerebral arteriosclerosis, reduce cerebral blood flow, and increase the risk of cognitive dysfunction by damaging nerve cell membranes and signal transduction mechanisms, as well as affecting the synthesis of steroid hormones and oxysterols (6, 7).

Elevated blood glucose level and dyslipidemia are closely related to cognitive dysfunction. Studies have found that elevated blood glucose level is linked to a higher risk of dementia in the elderly (8). Individuals with type 2 diabetes mellitus (T2DM) often experience dyslipidemia, characterized by elevated blood TC, triglycerides (TG), and LDL-C levels, and/or decreased HDL-C level (9). Dyslipidemia may aggravate cognitive dysfunction in T2DM patients (10). Therefore, compared with non-T2DM individuals, T2DM patients may have a higher risk of cognitive impairment (11, 12). In light of the T2DM status, and considering that chronic endocrine and metabolic abnormalities are closely related to cognitive function decline in T2DM patient (13), this cross-sectional study aims to reveal the relationship between plasma RC and cognitive function in T2DM and non-T2DM subjects, as well as the impact of dietary factors on it. Our findings will provide scientific data to support the development of precision nutritional strategies based on individual plasma RC levels, aimed at preventing MCI in middle-aged and elderly individuals, particularly among those with T2DM.

## 2 Methods

### 2.1 Participants

A total of 1817 adults aged 50 years and above were recruited from the Wulituo and Nanyuan communities, as well as Guang’anmen Hospital in Beijing, China. The participants underwent demographic and dietary surveys, biochemical index detection, and cognitive function assessment. Due to incomplete biochemical index measurements, 35 participants were excluded. Data from the remaining 1782 participants were finally included in the statistical analysis, with 675 diagnosed with MCI and 1,107 with normal cognitive function (Supplementary Figure S1). This study was conducted in accordance with the Declaration of Helsinki and was approved by the Medical Ethics Review Committee of Capital Medical University (No. 2012SY23). Written informed consent was obtained from all participants prior to the investigation.

### 2.2 Demographic characteristics

A self-designed questionnaire was used to investigate participants’ demographic characteristics (14). The information included basic characteristics [age, gender, education level (illiterate, primary school, junior high school, senior high school, junior college, undergraduate and above)], lifestyle factors [smoking (yes, no, abandon), alcohol drinking (yes or no), tea drinking (yes or no), living alone (yes or no), housework (yes or no), reading habits (yes or no), TV and computer (yes or no), usage of dietary supplements (yes or no), physical activity (never, 1–3 times/week, 4–5 times/week, daily)], and medical history of chronic disease [AD family history (yes or no), T2DM (yes or no), dyslipidemia (yes or no), chronic kidney disease (yes or no), cerebrovascular accident (yes or no)]. Nurses from the community health service centers measured the participants’ height and weight and calculated their body mass index [BMI = weight (kg)/height (m)^2^].

### 2.3 Dietary survey

The Food Frequency Questionnaire (FFQ) was utilized to evaluate individual daily dietary intake, including cereals, legumes, animal-based foods, nuts, vegetables, fruits, and cooking oil (15). The information gathered encompassed the frequency of consumption and the daily amount of food consumed, with participants’ dietary intake being calculated based on the size of their household. In line with prior research, the diet quality distance (DQD) was computed using data from the FFQ survey, serving as an indicator of overall dietary imbalance and was incorporated into the model to account for dietary factors (16).

### 2.4 Cognitive function measurement

The Chinese version of the MoCA scale was used for cognitive function measurement. The cut-off values of MoCA score for MCI were as follows: ≤13 for illiterate, ≤19 for participates with less than 6 years of education, and ≤24 for those with 7 or more than 7 years of education (14), which has been widely adopted to account for educational and cultural differences in Chinese populations. The test was conducted by doctors and trained nurses from the community health service center.

### 2.5 Biochemical measurements

Fasting peripheral venous blood (5 mL) was collected in the morning. After centrifuging at 3000 g for 15 min, the plasma was separated and stored in −80 °C refrigerators. Blood lipids including TC, LDL-C and HDL-C were measured using an automatic biochemical analyzer. Plasma RC concentration was calculated according to the formula: RC = TC concentration – (HDL-C concentration + LDL-C concentration) (17). All samples for each participant were analyzed within a single batch, and the inter-assay coefficients of variation (CV) were less than 5%.

### 2.6 Diagnostic basis of diabetes

T2DM was defined as an HbA1c level 6.5% or greater, fasting plasma glucose (FBG) level 7.0 mmol/L or greater, or self-reported having been diagnosed with T2DM by a hospital or community health service (18).

### 2.7 Statistical analysis

The statistical analyses were performed using IBM SPSS Statistics v.26.0 and R v.4.2.3. Graphs were created using R v.4.2.3 and GraphPad Prism 8. Continuous variables were expressed as mean ± standard deviation, and ANOVA was used for inter-group comparisons. Categorical variables were presented as *n* (%), with R × C χ^2^ tests applied for group comparisons. Participants were divided into 4 groups according to the quartiles of plasma RC concentrations: Q_1_ (0–0.349 mmol/L), Q_2_ (0.350–0.579 mmol/L), Q_3_ (0.580–0.849 mmol/L) and Q_4_ (above 0.850 mmol/L). The general linear model (GLM) was applied to compare differences of cognitive function and dietary intakes among groups. Logistic regression and restricted cubic spline (RCS) analyses were used to evaluate the association between plasma RC level and the risk of MCI. A trend test was conducted by incorporating the median quartile of RC into models. Subgroup analysis and mediation analysis were also conducted. Potential con-founders, including age, gender, BMI, education level, smoking status, alcohol consumption, tea intake, dietary supplement use, physical activity, living situation, household chores, reading habits, TV and computer usage, dyslipidemia, T2DM, cerebrovascular accident (CVA), chronic kidney disease (CKD), and family history of Alzheimer’s disease (AD), were adjusted during data analysis. Two-side *p*-value < 0.05 was considered statistically significant.

## 3 Results

### 3.1 Demographic characteristics, cognitive function, dietary intakes and plasma parameters

Table 1 presents the differences in demographic characteristics, lifestyle factors, and plasma parameters between the groups. Significant differences were observed in age, BMI, education level, reading habits, housework engagement, dietary supplement intake, histories of dyslipidemia, CVA and CKD between the groups (*p* < 0.05). Participants with T2DM and MCI had the highest plasma RC levels among the four groups (*p* = 0.001). Table 2 displays the differences in cognitive function and dietary intakes. Significant differences were observed in daily intakes of fruits, cereals, legume, fish, whole grains, red meat, nuts, eggs and milk intakes (*p* < 0.05). Participants with MCI showed lower scores on the total MoCA and in cognitive domains (*p* < 0.001).

**Table 1:** Demographic character and plasma parameters of the participants.

Variables	MCI		Non-MCI		*P-*value
	T2DM (*n* = 189)	Non-T2DM (*n* = 486)	T2DM (*n* = 300)	Non-T2DM (*n* = 807)	
Age (year)	67.91 ± 7.15	66.96 ± 6.63	65.99 ± 5.96	65.70 ± 5.83	<0.001
BMI (kg/m^2^)	25.49 ± 3.31	24.72 ± 3.30	25.27 ± 3.36	24.99 ± 3.41	0.026
Gender, *n* (%)					0.067
Male	74 (39.2)	165 (34.0)	114 (38.0)	252 (31.2)	
Female	115 (60.8)	321 (66.0)	186 (62.0)	555 (68.8)	
Education, *n* (%)					0.001
Illiterate	10 (5.3)	20 (4.1)	21 (7.0)	46 (5.7)	
Primary school	30 (15.9)	76 (15.6)	61 (20.3)	147 (18.2)	
Junior high school	90 (47.6)	235 (48.4)	116 (38.7)	283 (35.1)	
Senior High school	42 (22.2)	119 (24.5)	66 (22.0)	228 (28.3)	
Junior college	11 (5.8)	22 (4.5)	26 (8.7)	67 (8.3)	
Bachelor degree or above	6 (3.2)	14 (2.9)	10 (3.3)	36 (4.5)	
Physical activity, *n* (%)					0.082
Never	16 (8.5)	36 (7.4)	18 (6.0)	54 (6.7)	
1–3 times/week	15 (7.9)	40 (8.2)	29 (9.7)	96 (11.9)	
4–6 times/week	13 (6.9)	44 (9.1)	24 (8.0)	96 (11.9)	
Everyday	145 (76.7)	366 (75.3)	229 (76.3)	561 (69.5)	
Live alone, *n* (%)	19 (10.1)	32 (6.6)	26 (8.7)	43 (5.3)	0.054
Reading, *n* (%)	59 (31.2)	176 (36.2)	133 (44.3)	350 (43.4)	0.002
TV and computer use, *n* (%)	178 (94.2)	467 (96.1)	291 (97.0)	777 (96.3)	0.459
Housework, *n* (%)	167 (88.4)	444 (91.4)	273 (91.0)	774 (95.9)	<0.001
Smoking status					0.559
Yes, *n* (%)	27 (14.3)	62 (12.8)	46 (15.3)	119 (14.7)	
No, *n* (%)	136 (72.0)	371 (76.3)	213 (71.0)	603 (74.7)	
Abandon, *n* (%)	26 (13.8)	53 (10.9)	41 (13.7)	85 (10.5)	
Alcohol drinking, yes, *n* (%)	52 (27.5)	122 (25.1)	89 (29.7)	210 (26.0)	0.528
Tea drinking, yes, *n* (%)	105 (55.6)	263 (54.1)	173 (57.7)	452 (56.0)	0.802
Dietary supplement, yes, *n* (%)	49 (25.9)	133 (27.4)	82 (27.3)	166 (20.6)	0.016
Dyslipidemia, yes, *n* (%)	110 (58.2)	178 (36.6)	162 (54.0)	317 (39.3)	<0.001
AD family history, yes, *n* (%)	13 (6.9)	42 (8.6)	21 (7.0)	80 (9.9)	0.340
CVA, yes, *n* (%)	29 (15.3)	43 (8.8)	24 (8.0)	39 (4.8)	<0.001
CKD, yes, *n* (%)	23 (12.2)	31 (6.4)	22 (7.3)	32 (4.0)	<0.001
FBG (mmol/L)	7.82 ± 2.35	5.17 ± 0.49	7.39 ± 2.51	5.09 ± 0.59	<0.001
TC (mmol/L)	4.81 ± 1.13	5.12 ± 0.98	4.74 ± 1.10	5.05 ± 0.97	<0.001
TG (mmol/L)	1.89 ± 1.48	1.62 ± 0.99	1.94 ± 1.66	1.71 ± 1.17	0.001
LDL-C (mmol/L)	2.70 ± 0.96	2.96 ± 0.80	2.81 ± 0.94	2.98 ± 0.86	<0.001
HDL-C (mmol/L)	1.38 ± 0.29	1.47 ± 0.29	1.32 ± 0.27	1.44 ± 0.31	<0.001
RC (mmol/L)	0.73 ± 0.49	0.68 ± 0.38	0.61 ± 0.55	0.62 ± 0.42	0.001

Data was presented as mean ± standard or *n* (%). ANOVA was used to compare differences in age, BMI and plasma parameters; *χ*^2^ test was used to compare differences in gender, education, lifestyles and history of diseases between groups. *P* < 0.05 was considered statistically significant. BMI, body mass index; CVA, cerebrovascular accident; CKD, chronic kidney disease; AD, Alzheimer disease; T2DM, type 2 Diabetes Mellitus; TC, total cholesterol; TG, triglycerides; LDL-C, low-density lipoprotein cholesterol; HDL-C, high-density lipoprotein cholesterol; RC, remnant cholesterol.

**Table 2:** Cognition and dietary intake of participants.

Variables	MCI		Non-MCI		*P-*value
	T2DM (*n* = 189)	Non-T2DM (*n* = 486)	T2DM (*n* = 300)	Non-T2DM (*n* = 807)	
Cognitive function					
Visual and executive	2.71 ± 1.37	2.91 ± 1.36^a^	4.10 ± 1.00^ab^	4.12 ± 1.01^ab^	<0.001
Naming	2.69 ± 0.63	2.71 ± 0.66	2.94 ± 0.26^ab^	2.94 ± 0.29^ab^	<0.001
Attention	4.69 ± 1.58	4.80 ± 1.37	5.60 ± 0.80^ab^	5.60 ± 0.80^ab^	<0.001
Language	1.42 ± 0.83	1.31 ± 0.80	2.15 ± 0.80^ab^	2.26 ± 0.80^ab^	<0.001
Abstraction	0.98 ± 0.82	1.01 ± 0.75	1.69 ± 0.57^ab^	1.69 ± 0.60^ab^	<0.001
Memory and delayed recall	1.66 ± 1.47	1.65 ± 1.47	3.20 ± 1.36^ab^	3.34 ± 1.33^ab^	<0.001
Orientation	5.54 ± 1.14	5.43 ± 1.12^a^	5.91 ± 0.31^ab^	5.92 ± 0.34^ab^	<0.001
MoCA score	19.75 ± 4.83	19.87 ± 4.69	25.77 ± 2.72^ab^	26.01 ± 2.96^ab^	<0.001
Dietary					
Fruits intake (g/d)	138.06 ± 112.82	169.64 ± 113.03^a^	127.60 ± 94.40^b^	166.89 ± 114.60^ac^	<0.001
Vegetables intake (g/d)	307.67 ± 147.59	301.92 ± 140.31	322.83 ± 143.06	314.57 ± 137.31	0.109
Cereals intake (g/d)	260.16 ± 110.86	266.80 ± 116.51	247.09 ± 99.46^b^	261.93 ± 99.89^c^	0.006
Legume intake (g/d)	31.61 ± 35.98	37.45 ± 36.11^a^	30.51 ± 26.93^b^	32.42 ± 28.90^b^	0.008
Cooking oil intake (g/d)	33.34 ± 21.54	29.93 ± 23.24	30.14 ± 19.34	29.49 ± 16.23	0.126
Fish intake (g/d)	20.24 ± 23.97	23.03 ± 25.06	18.81 ± 16.60^b^	20.42 ± 18.65^b^	0.014
Whole grain intake (g/d)	45.96 ± 43.61	50.28 ± 47.52	35.25 ± 32.11^ab^	39.13 ± 39.71^b^	<0.001
Red meat intake (g/d)	31.60 ± 31.81	35.84 ± 35.75	30.98 ± 30.80^b^	31.19 ± 29.86^b^	0.032
Light meat intake (g/d)	14.95 ± 15.43	16.32 ± 20.77	14.11 ± 16.63	15.20 ± 16.96	0.265
Nut intake (g/d)	21.65 ± 28.26	23.04 ± 28.02	21.62 ± 38.15	17.96 ± 21.92^b^	0.016
Egg intake (g/d)	40.49 ± 25.87	41.26 ± 22.95	34.28 ± 20.89^ab^	35.62 ± 19.54^b^	<0.001
Milk intake (g/d)	146.42 ± 120.81	133.83 ± 101.36	139.27 ± 97.47	120.53 ± 105.87^ac^	0.004
DQD (points)	23.38 ± 7.02	22.04 ± 7.73	22.54 ± 7.45	21.92 ± 7.40	0.088

Data was presented as mean ± standard. General linear model and LSD test were used to compare differences in MoCA score and dietary intake between groups. Age, gender, BMI, education level, smoking status, alcohol and tea consumption, dietary supplement intake, physical exercise, living status, reading habits, TV and computer use, household chores, dyslipidemia, CVA, CKD, and family history of AD were adjusted for cognitive function comparison. Age, gender and BMI were adjusted for dietary intake comparison. ^a^Compared with T2DM-MCI group; ^b^compared with Non-T2DM-MCI group; ^c^compared with T2DM-Non-MCI group. *P* < 0.05 was considered statistically significant. T2DM, type 2 Diabetes Mellitus; CVA, cerebrovascular accident; CKD, chronic kidney disease; AD, Alzheimer disease; DQD, diet quality distance.

### 3.2 Correlation between plasma RC and MoCA score

**Figure 1:**
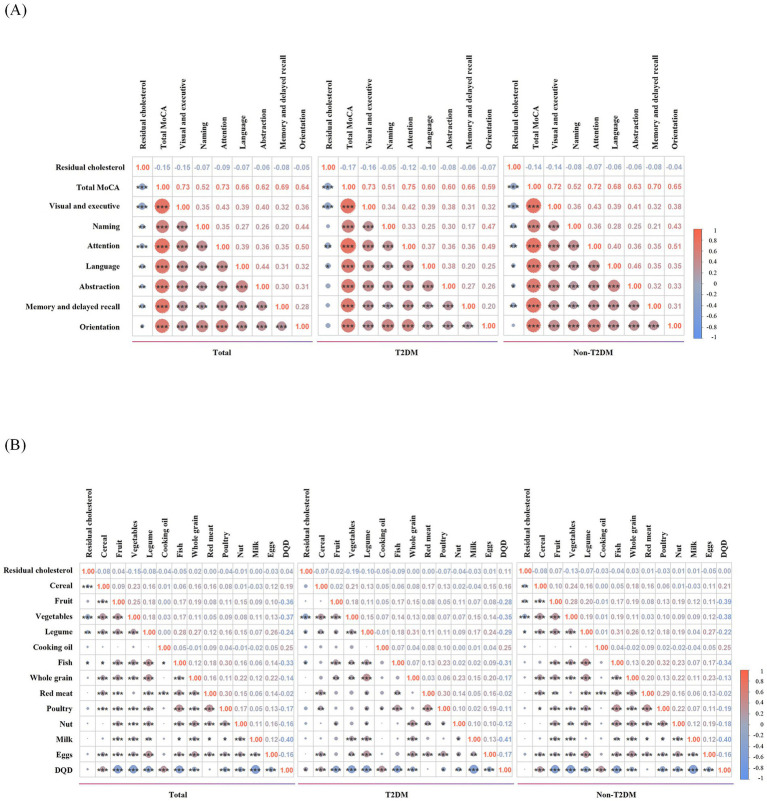
**(A)** Correlation between residual cholesterol and MoCA scores. **(B)** Correlation between residual cholesterol and dietary. *p < 0.05, **p < 0.01, ***p < 0.001.

As shown in Figure 1A, in the whole study population, plasma RC level was negatively correlated with total MoCA scores (*r* = −0.15, *p* < 0.001), as well as cognitive function in visual and executive (*r* = −0.15, *p* < 0.001), naming (*r* = −0.07, *p* < 0.01), attention (*r* = −0.09, *p* < 0.001), language (*r* = −0.07, *p* < 0.01), abstraction (*r* = −0.06, *p* < 0.01), memory and delayed recall (*r* = −0.08, p < 0.01), and orientation (*r* = −0.05, *p* < 0.05) domains. A similar trend was observed in non-T2DM individuals, except that no statistically significant association was found between plasma RC and orientation function (*p* > 0.05). In the T2DM patients, plasma RC was negatively correlated with total MoCA scores (*r* = −0.17, *p* < 0.001), and function in visual and executive (*r* = −0.16, *p* < 0.001), attention (*r* = −0.12, *p* < 0.01), and language (*r* = −0.10, *p* < 0.05) domains, while no significant association was observed in other cognitive domains (*p* > 0.05).

### 3.3 Correlation between plasma RC and the dietary intakes

In the T2DM group, plasma RC levels were negatively correlated with daily vegetable (*r* = −0.19, *p* < 0.001), legume (*r* = −0.10, *p* < 0.05) and fish intakes (*r* = −0.09, *p* < 0.05), but positively correlated with DQD levels (*r* = 0.11, *p* < 0.05). In the non-T2DM group, plasma RC levels exhibited a negative correlation with daily cereal (*r* = −0.08, *p* < 0.01), vegetable (*r* = −0.13, *p* < 0.001), and legume (*r* = −0.07, *p* < 0.05) intakes, but a positive correlation with daily fruit intake (*r* = 0.07, *p* < 0.01) (Figure 1B).

### 3.4 Association of plasma RC with the risk of MCI

**Figure 2:**
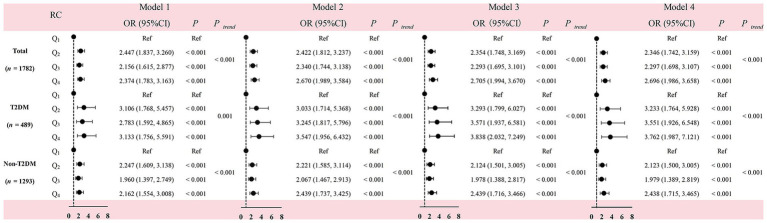
Association between residual cholesterol level and the risk of MCI in the participants with and without T2DM. Model 1: Unadjusted; model 2: adjusted for age, gender, BMI; model 3 was further adjusted for education level, smoking status, alcohol and tea consumption, dietary supplement intake, physical exercise, living status, reading habits, TV and computer use, household chores, dyslipidemia, CVA, CKD, and family history of AD; model 4 was further adjusted for individual’s DQD. Quartile of residual cholesterol level: Q1: 0–0.349 mmol/L; Q2: 0.350–0.579 mmol/L; Q3: 0.580–0.849 mmol/L; Q4: above 0.850 mmol/L. RC, residual cholesterol; MCI, mild cognitive impairment; CVA, cerebrovascular accident; CKD, chronic kidney disease; AD, Alzheimer disease; T2DM, type 2 diabetes mellitus; DQD, diet quality distance; OR: odds ratio; CI, confidence interval.

To accurately assess the relationship between plasma RC levels and the risk of MCI, four statistical models were applied (Figure 2). Before performing the formal analyses, we assessed multicollinearity among the independent variables using the variance inflation factor (VIF). Considering all 19 variables included in the models, the VIF values ranged from 1.009 to 1.952, indicating no significant multicollinearity. In the entire population, participants with Q_2_ (*OR* = 2.447, *p* < 0.001), Q_3_ (*OR* = 2.156, *p* < 0.001), and Q_4_ (*OR* = 2.374, *p* < 0.001) levels of plasma RC exhibited a higher risk of MCI compared to those with Q_1_ levels. Similarly, in the non-T2DM group, individuals with Q_2_ (*OR* = 2.247, *p* < 0.001), Q_3_ (*OR* = 1.960, *p* < 0.001), and Q_4_ (*OR* = 2.162, *p* < 0.001) levels of plasma RC also demonstrated a higher risk of MCI than those with Q_1_ levels. Participants with T2DM with Q_2_ (*OR* = 3.106, *p* < 0.001), Q_3_ (*OR* = 2.783, *p* < 0.001) and Q_4_ (*OR* = 3.133, *p* < 0.001) levels of plasma RC also exhibited an increased risk of MCI. These results remained consistent even after adjusting for multiple confounding factors in Models 2, 3 and 4.

A linear increasing trend the risk of MCI was observed from Q_1_ to Q_4_ group (*P*_trend_ < 0.001). A dose–response relationship was found between plasma RC and the risk of MCI. Additionally, the prevalence of MCI across the quartiles of plasma RC was observed.

**Figure 3:**
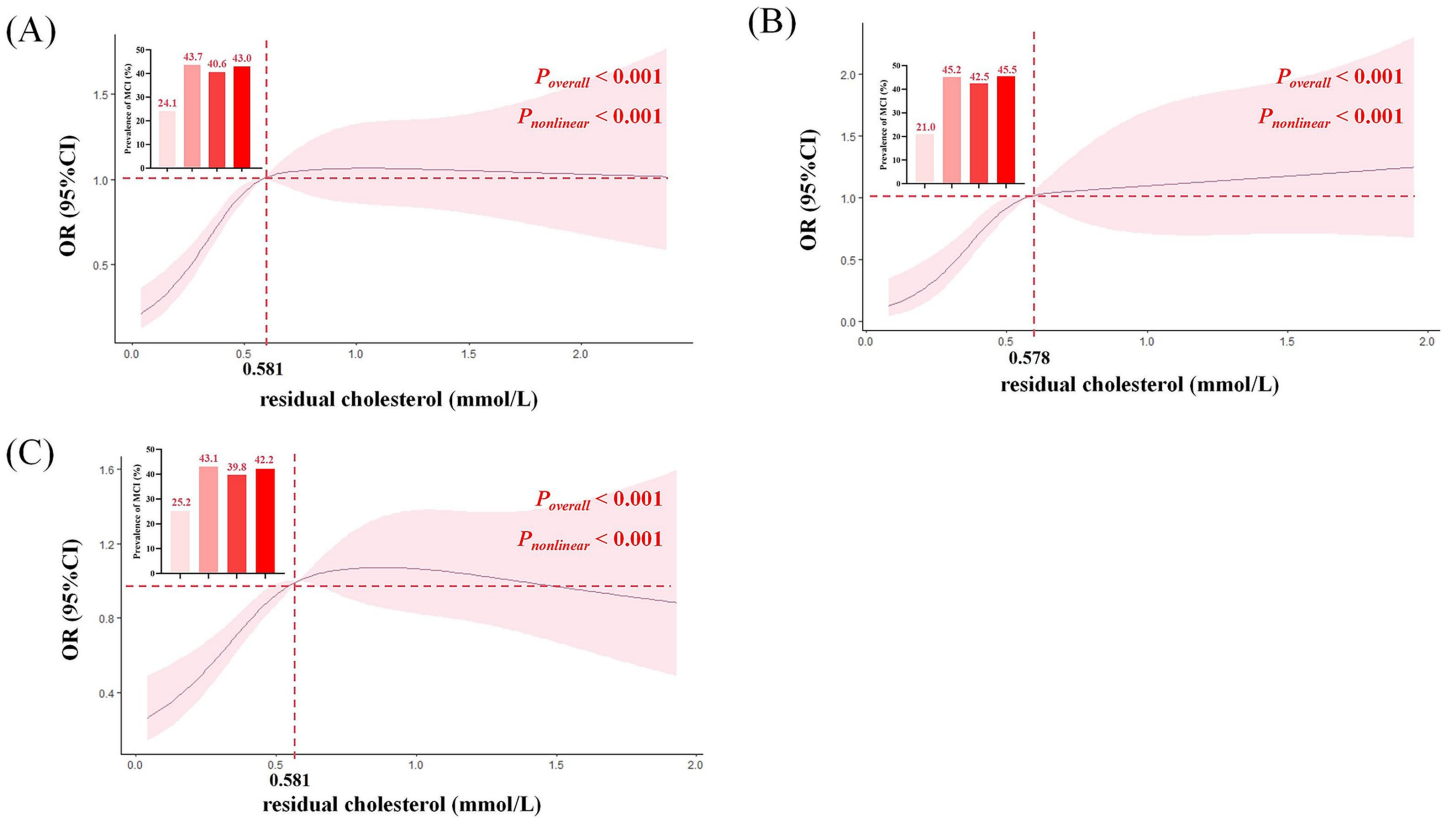
Restricted cubic splines to explore the relationship between residual cholesterol and the risk of MCI. **(A)** Total participants. **(B)** T2DM participants. **(C)** Non-T2DM participants. Multiple confounding factors including age, gender, BMI, education level, smoking status, alcohol and tea consumption, dietary supplement intake, physical exercise, living status, reading habits, TV and computer use, household chores, dyslipidemia, CVA, CKD, family history of AD and DQD. BMI, body mass index; CVA, cerebrovascular accident; CKD, chronic kidney disease; AD, Alzheimer disease; T2DM, type 2 diabetes mellitus; OR, odds ratio; CI, confidence interval.

As shown in Figure 3, the RCS analysis results revealed a nonlinear association between plasma RC level and the risk of MCI (*P*_overall_ < 0.001, *P*_non-linear_ < 0.001). Specifically, when plasma RC level was below 0.581 mmol/L, the risk of MCI increased sharply with increasing of plasma RC level. However, once plasma RC exceeded 0.581 mmol/L, the risk plateaued. The prevalence of MCI across the quartiles of plasma RC displayed similar patterns. In the overall population, the prevalence of MCI was lowest in the Q_1_ (24.1%) group, but significantly higher and relatively stable across the Q_2_ (43.7%), Q_3_ (40.6%), and Q_4_ (43.0%) groups. In T2DM participants, the prevalence of MCI across Q_1_ to Q_4_ groups was 21.0, 45.2, 42.5, and 45.5%, respectively. In non-T2DM participants, the MCI prevalence was 25.2, 43.1, 39.8, and 42.2% from Q_1_ to Q_4_ groups.

### 3.5 Subgroup analysis

**Figure 4:**
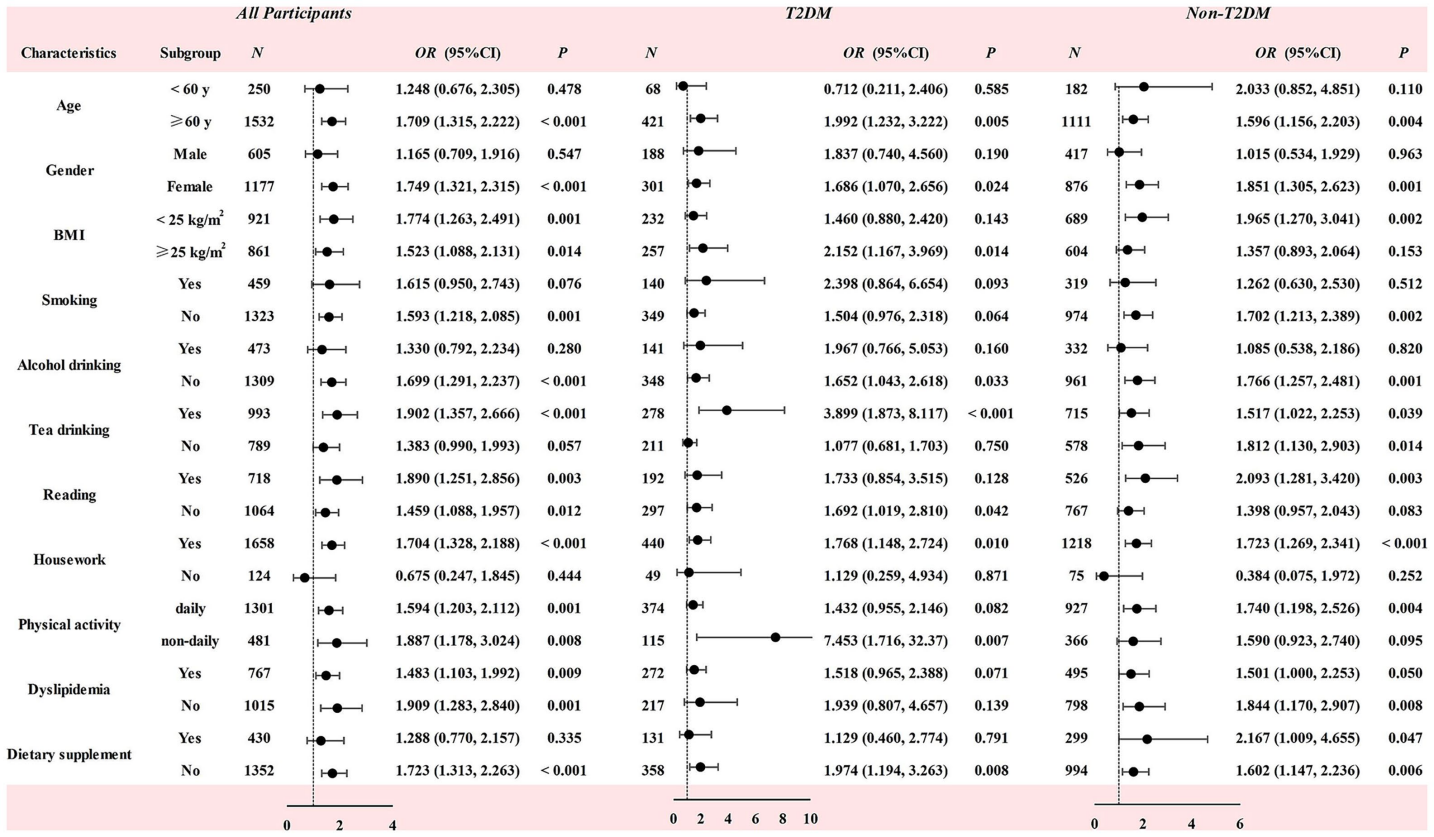
Association between residual cholesterol level and the risk of MCI in the participants with and without T2DM. Multiple confounding factors including age, gender, BMI, education level, smoking status, alcohol and tea consumption, dietary supplement intake, physical exercise, living status, reading habits, TV and computer use, household chores, dyslipidemia, CVA, CKD, family history of AD, and DQD. BMI, body mass index; CVA, cerebrovascular accident; CKD, chronic kidney disease; AD, Alzheimer disease; T2DM, type 2 Diabetes Mellitus; OR, odds ratio; CI, confidence interval. The adjustment for confounding factors did not include the stratification factors themselves.

The association between plasma RC level and risk of MCI across various subgroups was shown in Figure 4. In the whole population, a significantly positive correlation was observed between plasma RC level and the risk of MCI, particularly among females (*OR* = 1.749, *p* < 0.001), individuals aged 60 years and above (OR = 1.709, *p* < 0.001), non-smokers (OR = 1.593, *p* = 0.001), non-drinkers (OR = 1.699, *p* < 0.001), those drinking tea (OR = 1.902, *p* < 0.001) and not taking dietary supplements (OR = 1.723, *p* < 0.001), and individuals engaging in daily housework (OR = 1.704, *p* < 0.001). Within the T2DM group, the positive correlation between plasma RC and the risk of MCI was statistically significant among females (OR = 1.686, *p* = 0.024), individuals aged 60 years and above (OR = 1.992, *p* = 0.005), individuals with BMI ≥ 25 kg/m^2^ (OR = 2.152, *p* = 0.014), non-drinkers (OR = 1.652, *p* = 0.033), tea drinkers (OR = 3.899, *p* < 0.001), individuals without a reading habit (OR = 1.692, *p* = 0.042), those doing housework (OR = 1.768, *p* = 0.010), as well as those not taking dietary supplements (OR = 1.974, *p* = 0.008) showed the statistical significance. In the non-T2DM group, the females (OR = 1.851, *p* = 0.001), individuals aged 60 years and above (OR = 1.596, *p* = 0.004), those with BMI < 25 kg/m^2^ (OR = 1.965, *p* = 0.002), individuals without smoking habit (OR = 1.702, *p* = 0.002), non-alcohol drinkers (OR = 1.766, *p* = 0.001), individuals with a reading habit (OR = 2.093, *p* = 0.003), those doing housework (OR = 1.723, *p* < 0.001), and those doing daily physical exercise (OR = 1.740, *p* = 0.004) was mainly observed.

### 3.6 Mediation analysis

**Figure 5:**
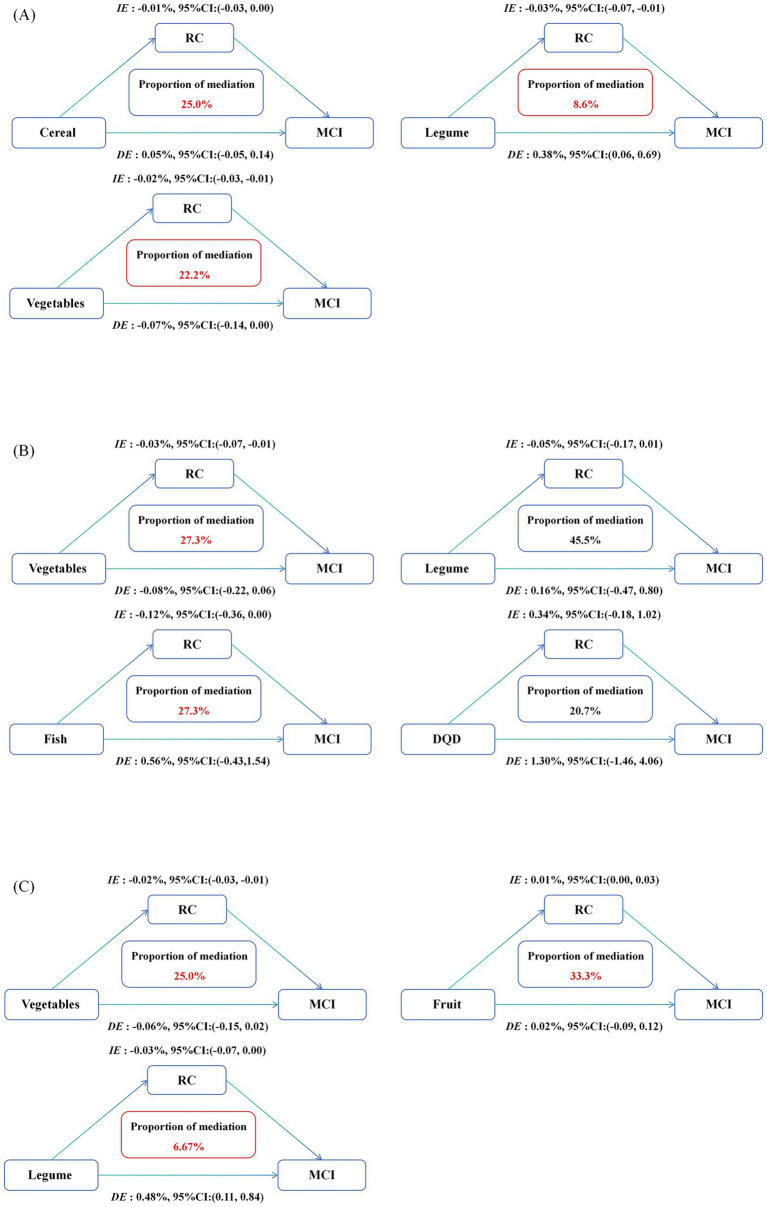
Estimated proportion of the association between dietary intakes and MCI mediated by plasma RC in the whole population **(A)**, T2DM population **(B)**, and non-T2DM population **(C)**. Models were adjusted for age, gender, BMI, education level, physical exercise, living status, reading habits, TV and computer use, household chores, smoking status, alcohol and tea consumption, dietary supplement intake, dyslipidemia, CVA, CKD, and family history of AD. IE, the estimate of the indirect effect; DE, the estimate of the direct effect; proportion of mediation = IE/DE + IE.

We further conducted a mediation analysis with plasma RC as the mediator (Figure 5). In the whole population, plasma RC significantly mediated the relationship between the intakes of cereal, vegetables, legumes and MCI, accounting for 25.0, 22.2 and 8.6%, respectively. In participants with T2DM, plasma RC mediated the association between vegetable and fish intake and MCI, accounting for 27.3% for both. In the participants without T2DM, significant mediation effects were observed for vegetables, fruits, and legumes, accounting for 25.0, 33.3, and 6.67% of the associations with MCI, respectively.

## 4 Discussion

In our study, we identified a positive correlation between plasma RC level and the risk of MCI in the T2DM and non-T2DM participants. Furthermore, we explored threshold of the plasma RC levels for predicting MCI risk and assessed the impact of dietary factors and lifestyles on the relationship between plasma RC levels and the risk of MCI.

Previous population-based cross-sectional studies have showed that elevated plasma TC is a risk factor for cognitive decline, and data from cohort study further demonstrated that higher plasma TC level in midlife significantly increase the risk of late-life MCI and AD (19, 20). However, the associations of other lipids, including TG, LDL-C, and HDL-C, with cognition remain controversial. A meta-analysis of 25 prospective studies reported that elevated plasma TG level is closely related to an increased risk of MCI, with every 3 mmol/L increase in TG concentration, the risk of MCI rises by 12% (21). He et al. (22) and Yin et al. (23) reported a negative association between plasma TG level and the risk of MCI. An increase of 1 mmol/L in serum LDL-C has been linked to a 53% lower risk of dementia (24). However, other studies reported inconsistent results (25). Notably, a study conducted by Hua indicated that plasma LDL-C level below 70 mg/dL exerts a protective effect on cognitive function (26). Lee’s study found a positive correlation between blood HDL-C level and cognitive impairment in AD patients (27). However, a community-based case–control study in China indicated that plasma HDL-C level above 1.04 mmol/L is significantly associated with a reduced risk of MCI (28). Findings from other studies further suggested that decreased plasma HDL-C level may be a risk factor for cognitive decline (29, 30).

The contradictory conclusions derived from different studies suggest that traditional lipid parameters may not be reliable indicators for the prediction of MCI. Our study identified plasma RC as a more stable and direct predictor of MCI risk, demonstrating a significant positive correlation with the risk of MCI. In the entire population, participants with Q_2_, Q_3_, and Q_4_ levels of plasma RC had an increased risk of MCI compared to those with Q_1_ levels. Consistent with our findings, a nationwide population-based cohort study in South Korea found that higher plasma RC concentration was independently associated with an increased risk of all-cause dementia, including AD and vascular dementia (17). Besides, the negative association between plasma RC and cognitive function remains significant in the US population (6). A cross-sectional study included 36 patients with MCI and 38 non-MCI also found that MCI patients had a higher level of plasma RC (31). Due to vascular injury and cerebrovascular disease, deregulation of insulin signaling in the brain, inflammation and oxidative stress, and abnormal glucose metabolism, T2DM patients are often at higher risk of cognitive decline (32, 33). Thus, we further explored the relationship of plasma RC and cognition in T2DM and non-T2DM subjects. In the non-T2DM group, compared with those with Q_1_ levels of plasma RC, subjects with Q_2_ (*OR* = 2.247, *p* < 0.001), Q_3_ (*OR* = 1.960, *p* < 0.001), and Q_4_ (*OR* = 2.162, *p* < 0.001) level of plasma RC exhibited a higher risk of MCI. In the T2DM group, subjects with Q_2_ (*OR* = 3.106, *p* < 0.001), Q_3_ (*OR* = 2.783, *p* < 0.001) and Q_4_ (*OR* = 3.133, *p* < 0.001) levels of plasma RC also exhibited a higher risk of MCI. Different risks (OR values) were observed between the T2DM and non-T2DM subjects, with the T2DM subjects displaying much higher OR value than the non-T2DM subjects (3.106 vs. 2.247, 2.783 vs. 1.960, and 3.133 vs. 2.162). An increase of plasma RC level predisposes the T2DM subjects to a much higher risk of MCI than the non-T2DM subjects. These results are consistent with previous studies (17), which reported that high level of plasma RC was associated with an increased risk of all-cause dementia in subjects with diabetes. All these data indicated the importance of lipid control in preventing cognitive decline in subjects with T2DM.

Our study revealed that the association between plasma RC and MCI was dependent on gender, age, and lifestyle. This was demonstrated by the non-significant correlation in male subjects, those aged under 60, alcohol consumers, individuals not engaging in household chores, and users of dietary supplements. The gender-discrepancy may be due to the differences in hormone levels between the male and the females. A rapid decline in estrogen levels after menopause in women can alter cholesterol metabolism and diminish its neuroprotective effects (34). In addition, we did not find this relationship in participants aged under 60 years. Other studies reported the different results, and the relationship of plasma RC concentration and the risk of dementia was more prominent in the middle age subjects compared to the older ones. The small sample size of the middle-aged group might contribute to the different results between studies. Moreover, we found the relationship was statistically significant in the subjects without alcohol drinking habit. This result is similar to findings from a study of alcohol consumption and cognitive function, which reported that subjects with small daily amounts of alcohol consumption had a lower risk of impaired cognitive function compared to those who did not consume alcohol (35). However, the relationship was not significant in subjects using dietary supplements, possibly because these supplements may mitigate the risk of cognitive decline, thereby obscuring the relationship between plasma RC and MCI (36).

We also found that the prevalence of MCI in Q_2_, Q_3_, and Q_4_ groups is higher than that in Q_1_ group. However, the prevalence of Q_2_, Q_3_, and Q_4_ groups are similar. Results of RCS analysis also suggested that the risk of MCI and plasma RC was non-linear (*P*_overall_ < 0.001, *P*_non-linear_ < 0.001). The risk of MCI increased sharply when plasma RC level was below 0.581 mmol/L, and stabilized at the concentration above 0.581 mmol/L in the whole population and in the non-T2DM subjects. For the T2DM subjects, the risk of MCI increased sharply when RC level was below 0.578 mmol/L. This cut off value is close to the plasma RC level for decreasing the risk of MCI in the whole population. These results suggest that a relatively lower plasma RC threshold, possibly lower than the 0.80 mmol/L reported in a previous study (37), might be recommended for T2DM subjects to reduce the risk of MCI.

Besides, we found that plasma RC level was closely related to dietary intakes. Previous studies have showed that dietary fiber and phytosterols in plant foods, such as whole grains, soybeans, and vegetables, could lower serum cholesterol level through affecting intestinal cholesterol absorption (38). Intakes of roots, stems and leaves of vegetables have been demonstrated associated with lower VLDL cholesterol level (39). Intake of unsaturated fatty acids, such as Omega-3 fatty acids from fish, may lower triglyceride level by inhibiting VLDL triglyceride synthesis (40). Consistently, in our study, we found that plasma RC level was negatively correlated with daily vegetable, legume, and fish intake in the T2DM group. In the non-T2DM group, plasms RC level exhibited a negative correlation with daily cereal, vegetable, and legume intakes. The discrepant relationship between diets and plasma RC in T2DM and non-T2DM subjects might be attributable to the self-management of dietary pattern to rescue abnormal glucose and lipids metabolism in the T2DM patients.

Our study has several strengths. It is the first to identify appropriate RC values that can reduce the risk of MCI in middle-aged and elderly Chinese individuals. This association is more pronounced in those with T2DM, where the RC risk threshold is lower compared to non-T2DM individuals. Unlike traditional lipid markers, RC serves as an effective and stable predictor of MCI. These findings have significant implications for the early warning and screening of high-risk populations for MCI. Furthermore, dietary modifications guided by RC risk thresholds may help mitigate cognitive decline.

There are some limitations in this study. Firstly, the cross-sectional design hinders our ability to dynamically monitor the persistent impact of plasma RC on cognition and the risk of MCI in participants, resulting in an inability to infer causality. Therefore, longitudinal studies are necessary in the future to explore the temporal changes and causal relationships of these connections. Secondly, the sample size in our study is relatively small, and the population is limited to one city, which may introduce potential biases. Although we have controlled some potential confounding factors, others may potentially affect cognitive ability and the risk of MCI. For example, genetic predisposition, socioeconomic status, and access to healthcare service might also differentially impact on an individual’s cognitive function. Future research should consider more potential factors to comprehensively understand the relation of plasma RC with cognitive decline and the risk of MCI. Thirdly, this study did not incorporate biological markers of vascular injury, inflammation, or oxidative stress, nor did it include neuroimaging data, such as brain MRI. As a result, we were unable to directly verify the potential intermediate mechanisms or structural brain changes through which plasma RC may contribute to cognitive impairment. Furthermore, plasma RC levels were estimated using a validated calculation-based approach rather than direct measurement techniques such as ultracentrifugation or nuclear magnetic resonance. Although this approach has been widely used in epidemiological studies, future studies employing direct assay methods would help minimize potential measurement bias and improve the robustness of the findings. Future research should consider more potential factors to comprehensively elucidate the relation of plasma RC with cognitive decline and the risk of MCI.

## 5 Conclusion

Elevated plasma RC level is a potential risk factor for MCI, with protective thresholds below 0.578 mmol/L for T2DM and below 0.581 mmol/L for non-T2DM subjects, which can reduce the risk of MCI in middle-aged and elderly individuals with and without T2DM, respectively. Optimal daily intakes of vegetables, legumes and fish may help control plasma RC level and protect cognitive function in T2DM patients. Future studies should include larger sample sizes and account for potential confounding factors to validate our findings.

## Data Availability

The datasets are not publicly available due to confidentiality and controlled access policies. Anonymized data may be obtained from the corresponding author upon reasonable request.
