# Supplementary figures and images for: Association of plasma remnant cholesterol with cognitive function in the middle-aged and elderly Chinese adults with type 2 diabetes: a cross-sectional study
Source: Front Nutr. 2026 Feb 5;13:1705243. doi: 10.3389/fnut.2026.1705243 (PMC12922238; doi:10.3389/fnut.2026.1705243)

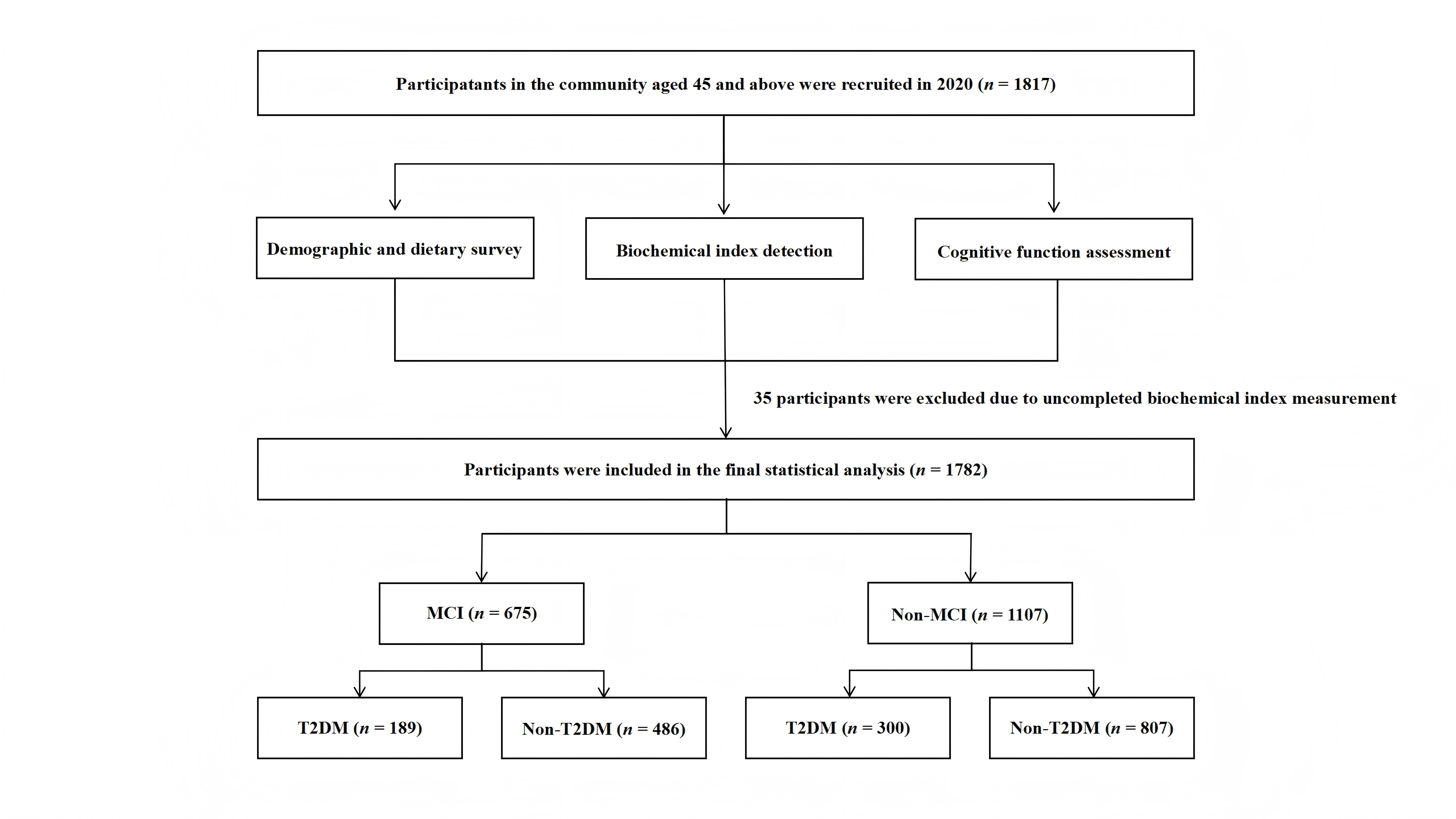

Supplement: Supplementary file 1 [file Image_1.jpeg]
